# Surveillance for *Neisseria meningitidis* Disease Activity and Transmission Using Information Technology

**DOI:** 10.1371/journal.pone.0127406

**Published:** 2015-05-20

**Authors:** S. Sohail Ahmed, Ernesto Oviedo-Orta, Sumiko R. Mekaru, Clark C. Freifeld, Gervais Tougas, John S. Brownstein

**Affiliations:** 1 Global Clinical Sciences, Novartis Vaccines Srl, Siena, Italy; 2 Children’s Hospital Informatics Program at the Harvard-Massachusetts Institute of Technology Division of Health Sciences and Technology, Computational Epidemiology Group, Boston, Massachusetts, United States of America; 3 Drug Safety and Epidemiology, Novartis Pharma AG, Basel, Switzerland; 4 Department of Pediatrics, Harvard Medical School, Boston Children’s Hospital, Boston, Massachusetts, United States of America; University of Waterloo, CANADA

## Abstract

**Background:**

While formal reporting, surveillance, and response structures remain essential to protecting public health, a new generation of freely accessible, online, and real-time informatics tools for disease tracking are expanding the ability to raise earlier public awareness of emerging disease threats. The rationale for this study is to test the hypothesis that the HealthMap informatics tools can complement epidemiological data captured by traditional surveillance monitoring systems for meningitis due to *Neisseria meningitides* (*N*. *meningitides*) by highlighting severe transmissible disease activity and outbreaks in the United States.

**Methods:**

Annual analyses of *N*. *meningitides* disease alerts captured by HealthMap were compared to epidemiological data captured by the Centers for Disease Control’s Active Bacterial Core surveillance (ABCs) for *N*. *meningitides*. Morbidity and mortality case reports were measured annually from 2010 to 2013 (HealthMap) and 2005 to 2012 (ABCs).

**Findings:**

HealthMap *N*. *meningitides* monitoring captured 80-90% of alerts as diagnosed *N*. *meningitides*, 5-20% of alerts as suspected cases, and 5-10% of alerts as related news articles. HealthMap disease alert activity for emerging disease threats related to *N*. *meningitides* were in agreement with patterns identified historically using traditional surveillance systems. HealthMap’s strength lies in its ability to provide a cumulative “snapshot” of weak signals that allows for rapid dissemination of knowledge and earlier public awareness of potential outbreak status while formal testing and confirmation for specific serotypes is ongoing by public health authorities.

**Conclusions:**

The underreporting of disease cases in internet-based data streaming makes inadequate any comparison to epidemiological trends illustrated by the more comprehensive ABCs network published by the Centers for Disease Control. However, the expected delays in compiling confirmatory reports by traditional surveillance systems (at the time of writing, ABCs data for 2013 is listed as being provisional) emphasize the helpfulness of real-time internet-based data streaming to quickly fill gaps including the visualization of modes of disease transmission in outbreaks for better resource and action planning. HealthMap can also contribute as an internet-based monitoring system to provide real-time channel for patients to report intervention-related failures.

## Introduction

*Neisseria meningitidis* (*N*. *meningitidis*) is a human pathogen that spreads between individuals through direct contact with respiratory secretions [1]. The bacteria can cause septicemia, pneumonia, and meningitis. *N*. *meningitidis* strains can be categorized, based on the composition of the bacteria’s capsule, into 13 different serogroups of which five are responsible for the great majority of invasive disease (serogroups A, B, C, W, and Y) [2]. Up to 14% of meningococcal infected individuals die [2,3] and among the survivors, up to 20% have serious sequelae including hearing loss, limb amputation, and neurologic disability [4]. Due to the disease’s rapid progression (< 24–48 hours), serious sequelae, and potential for person-to-person spread, a single case of meningococcal disease will trigger immediate and increasing media attention during which public health officials are working to avoid panic, to identify whether there is an outbreak, and to generate an effective response to treat disease and prevent further transmission.

Recently, two United States (US) universities have experienced serogroup B strain outbreaks that were widely reported by both traditional media and internet-based news reporting. The widespread adoption of more sophisticated information technology has increased public awareness of potentially serious diseases as seen during the emergence and global spread of the 2009 pandemic influenza A/H1N1 virus [5]. While formal reporting, surveillance, and response structures remain essential to protecting public health, a new generation of online, freely accessible, and real-time disease surveillance-monitoring tools have become available. These tools complement and expand existing public health professionals’ capacities to detect weak signals and to raise earlier concerns regarding emerging diseases [6–8]. In this report, we used the HealthMap informatics tool [9] to investigate the burden of “meningitis” that can be captured in the US. We aim to test the hypothesis that HealthMap complements data captured by traditional surveillance and provides insights related to the mode of disease transmission linked to emerging outbreaks.

## Methods

### HealthMap

HealthMap (http://www.healthmap.org/) is a multistream real-time surveillance platform that continually aggregates reports on new and ongoing infectious disease outbreaks. The system performs extraction, categorization, filtration, and integration of these reports, facilitating knowledge management and earlier awareness of potential outbreak status [10].

#### HealthMap system capabilities

The HealthMap automated text processing system proceeds in four stages: (1) Acquisition (2) Characterization (3) Filtering, and (4) Clustering. Trained public health analysts are employed to review content and correct misclassifications. Finally, the correctly classified content is used to improve the automated algorithms through a training feedback loop.

The first step is data acquisition. The system collects data via five main channels: news aggregators (such as Google News, Yahoo News, Moreover); specific Real-time Simple Syndication (RSS) feeds including WHO (World Health Organization), OIE (Office International des Epizooties/World Organisation for Animal Health), and others; e-mail subscriptions (GPHIN [Global Public Health Intelligence Network (developed by Health Camada)], ProMED, etc); custom-parsed HTML (HyperText Markup Language) scraping or CSV (Comma-separated Values)/XML (Extensible Markup Language) feeds; and user submitted reports. The framework allows one to add a new feed through a custom PHP (Personal Home Page Tools) Hypertext Preprocessor class where the feed-specific code fetches the content, parses it, and then extracts a set of common fields.

In the second step, the common outputs of the feed framework then pass to the characterization engine where we extract disease and location entities, flag the report as not-disease-related by rule (if applicable), and identify the source publication. This module loads large dictionaries of terms into memory and then matches them against the input text using a rapid matching algorithm. Algorithm performance is linear on the size of the input text and memory consumption is linear on the size of the dictionary. Under some conditions, the characterization module may make calls back to the acquisition module to request additional input text from the source. The dictionaries currently support identification of about 250 diseases. HealthMap has an internal location database of over 25,000 locations. The characterization engine generally assigns countries or first level administrative units; analysts generally change these to cities, hospitals, or schools, if that level of specificity is warranted.

Once disease and location are assigned, the document then passes to a document-level Bayesian filtering module (third step). The filter breaks the document into unigrams, digrams, trigrams (and tetragrams in some languages), and then computes a likelihood score based on training data from tens of thousands of previously recorded documents. It then assigns the document to one of five categories based on relevance; documents falling in the two most relevant categories, Breaking News and Warning, are posted to the map, whereas documents in the Old News, Context, or Not Disease Related categories are hidden from the map. These alerts are retained in the database for use in future research.

The last step in the automated pipeline is document clustering to group duplicate reports together. The clustering module compares the given document to each previously collected document over a fixed time window, and computes a similarity score based on the texts and classification outputs. If the maximum score is above a set threshold, the new document is assigned as a “child” of the previous document, and hidden from the map. The similarity calculation is based on six factors: (1) character-level headline comparison; (2) word-level headline comparison (fraction of words in common, with inverse term frequency taken into account); (3) disease and place comparison from the output of the named-entity extraction engine; (4) country-level comparison (i.e., if the two reports have different locations within the same country) boost similarity score somewhat; (5) timestamp (reports closer in time are more likely to be similar); and (6) filter category comparison based on the output of the Bayesian filtering module.

Following automated processing, HealthMap alerts are posted directly to the public map, partner pages, and RSS feeds. However, at the same time, a team of trained analysts will review all incoming data and correct misclassifications and assign additional higher resolution metadata classifications. For these curation tasks, a feature-rich analyst dashboard allows users to search, review and annotate the alerts. For example, analysts can highlight text in a report and assign a named entity category such as executing a geocoding query with the highlighted text and bringing up a map for use in assigning precise location to a report. The analyst corrections feed back into the machine learning algorithms and improve the automated content analysis. Case counts data is entered and additional tags are assigned when relevant. The current list of tags includes such diverse topics as schools, healthcare facilities, sports-associated, recall (of a product), and cruise.

#### HealthMap data sources

HealthMap uses a large variety of inputs to detect outbreak news displayed on the main web page, to produce password protected sites for partners with sensitive data, and to do research on novel disease surveillance methods.

The standard data inputs for the main webpage are: news aggregators (such as Google News, Yahoo News, Baidu), specific RSS feeds (WHO, OIE, FAO), constructed datasets of language-specific RSS feeds, email subscriptions (GPHIN, ProMED, etc), custom-parsed HTML scraping or CSV/XML feeds, and user submitted reports. HealthMap queries news aggregators at regular intervals with a list of queries designed to return outbreak news stories with excellent sensitivity while avoiding poor specificity. Post-collection processing ensures non-outbreak related articles are not displayed on the map. Specific RSS feeds and e-mail subscriptions allow the system to receive high quality data from known providers. Custom-parsed scraping programs allow the system to retrieve data that is otherwise difficult to access.

User-submitted reports represent a diverse group of resources. Users may submit URLs or eyewitness descriptions of outbreaks to HealthMap via the website, e-mail, telephone, text message or the Outbreaks Near Me mobile app for Android and iPhone. These reports are processed through various means depending on the method of delivery.

Social media sources are also included in HealthMap. Twitter and Facebook data are collected and processed by the parser. The short nature of tweets does present challenges to automated categorization, but enhanced dictionaries have helped to address that issue.

#### HealthMap subject matter domains

Although HealthMap was developed specifically to provide a comprehensive real time display of infectious disease outbreak knowledge, the system has proven flexible and robust enough to support many other projects. HealthMap has been used in diverse government-sponsored and academic research-oriented projects including monitoring violence against civilians in the Syrian conflict, and enhancing situation awareness for response efforts for a natural disaster. The core architecture and processing capabilities are language and subject matter independent. HealthMap is constantly expanding its language capabilities. The core HealthMap languages have extensive language support: English, Spanish, Portuguese, French, Russian, Chinese, and Arabic. More recently added language capabilities are German, Korean, Japanese, Vietnamese, Malay and Indonesian. HealthMap also processes alerts in Italian and Thai for specific projects. HealthMap aims to continue to adding language capabilities to further expand its global coverage of hyper-local outbreaks. The relatively straightforward process for adding languages demonstrate the flexibility of the underlying automated parser.

### Study design and population

#### HealthMap data capture and analyses

HealthMap became operational for *N*. *meningitidis* disease capture in 2010 and was used for this study to detect cases of meningococcal disease and potential outbreaks in the US from 2010 to 2013. Heat maps were generated using the following search conditions: meningitis-*Neisseria*, United States, new & ongoing outbreaks, and yearly intervals. Alerts reflect online information from news, expert-curated discussions, and validated official reports (e.g., World Health Organization, ProMED Mail, and Google News feeds that were active during this time interval). Alerts were individually crosschecked manually to ensure they reflected confirmed cases of meningococcal disease with citation of the appropriate state health departments or institutions. Accuracy of HealthMap capture was calculated based on downloading all the citations and individually counting the alerts to verify those capturing diagnoses, suspected cases, or general news.

An “alert” in HealthMap is defined as any unique data source, typically a unique URL link. If the data source (i.e. Associated Press or Reuters) writes a story and 30 newspapers pick it up and write slightly different versions, then the system receives all of those URLs as distinct alerts, but the artificial intelligence of the system will group them together as having identical information despite existing in different locations in the Internet. The term alert is used sometimes interchangeably as the presence of a case, but occasionally it could also be a warning.

#### Centers for Disease Control data capture and analyses

Traditional surveillance data were obtained from the Centers for Disease Control (CDC) Active Bacterial Core surveillance (ABCs) Emerging Infections Program Network [11] for *N*. *meningitidis* from 2005 (to include assessment of the impact of vaccine introduction on disease incidence) to 2012 (most recent finalized report available online). Non-B serogroup and serogroup B case rates per 100,000 population reflects ABCs figures in ten US states representing 39 to 43 million inhabitants over eight years. Total cases in the US reflect CDC estimates based on applying ABC case rate to US population census.

## Results

### The HealthMap meningitis system—increasing visibility of meningococcal cases

#### Geographic distribution of HealthMap alerts in the US

**Fig 1:**
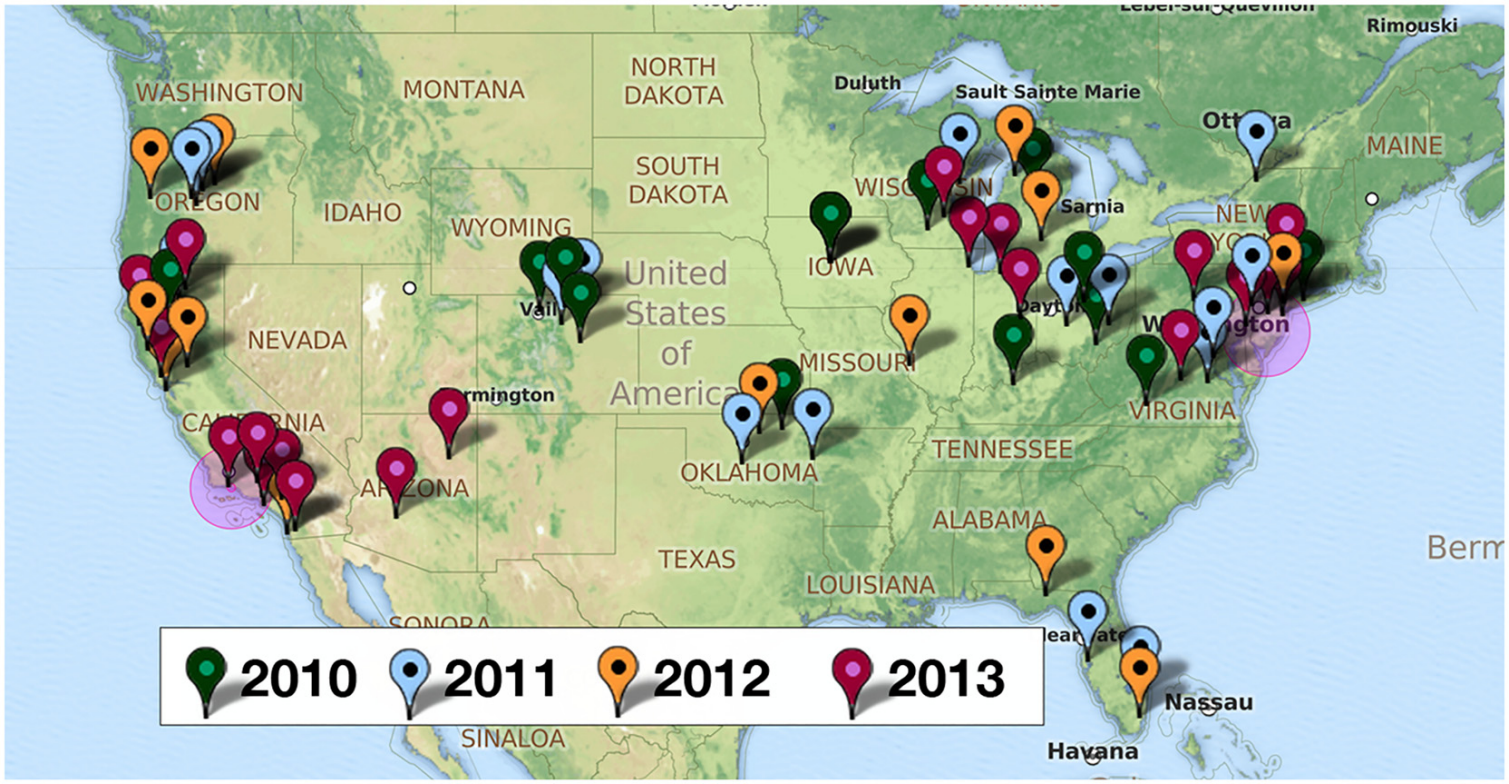
HealthMap data for Neisseria meningitidis alerts from 2010 to 2013. Markers indicate state, province, or local alerts. The two pink-shaded circles represent country-level alerts for 2013 due to the serogroup B outbreaks at University of California Santa Barbara and Princeton University. Image generated using ZeeMaps. Reprinted under a CC BY license with permission from ZeeMaps.

**Fig 2:**
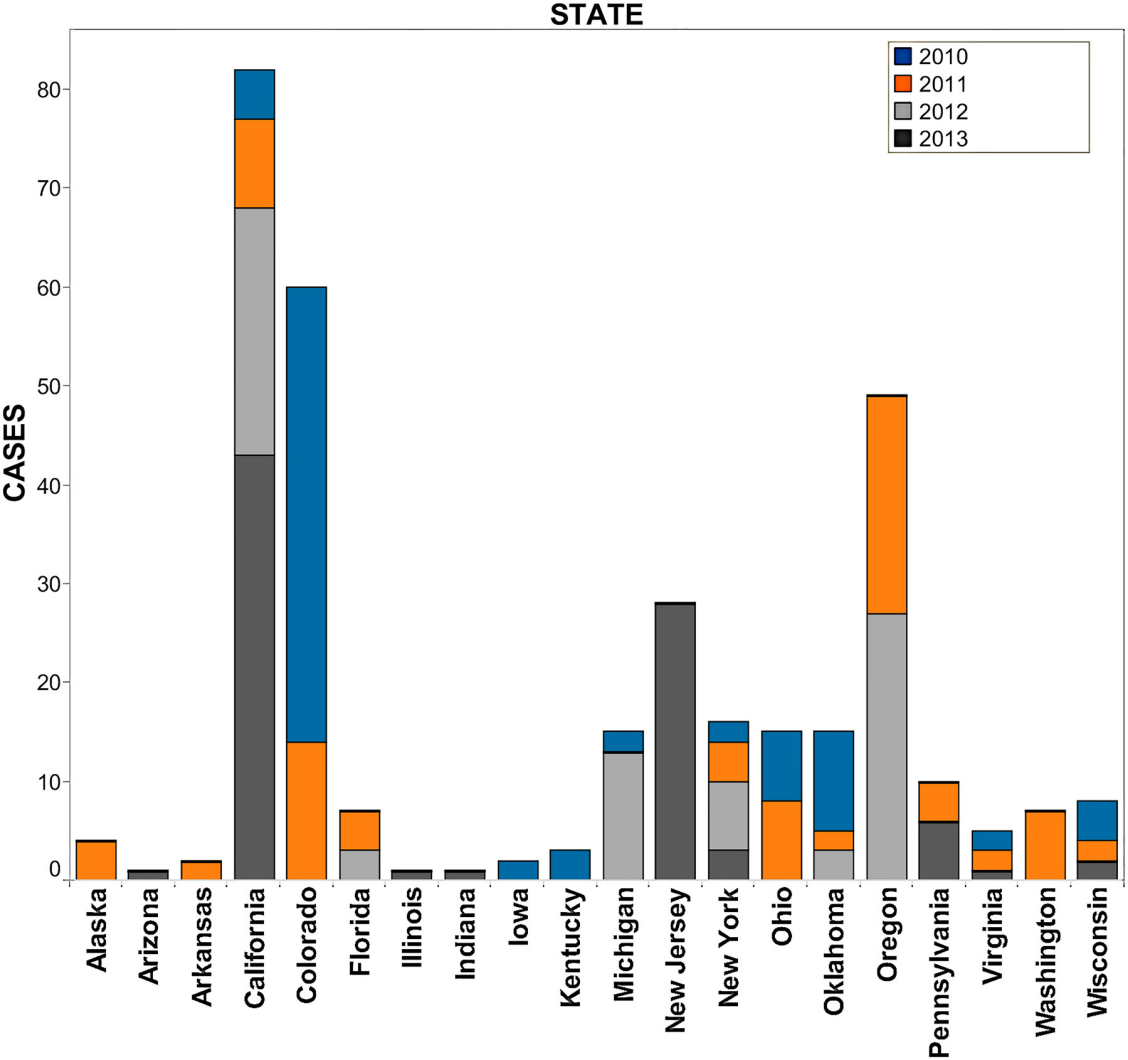
Number of HealthMap meningococcal case alerts in twenty US states reporting disease activity from 2010–2013. The majority of internet-based case alerts derived from meningococcal disease activity in Oklahoma/Colorado (12.0% and 55.4% in 2010), Colorado/Oregon (16.7%/26.2% in 2011), Oregon/California (34.6% and 32.1% in 2012), and California/New Jersey (50.0% and 32.6% in 2013). Bars indicate actual number of alerts. Blue color indicates case alerts in 2010, orange color indicates case alerts in 2011, light grey color indicates case alerts in 2012, and dark grey color indicates case alerts in 2013.

Fig 1 illustrates the distribution of HealthMap meningococcal alerts from 2010–2013 that are color-coded by year. The two pink shaded circles represent country levels alerts indicated by HealthMap for 2013 due to the serogroup B outbreaks at Princeton University and University of California Santa Barbara. Compared to a retrospective surveillance study reviewing outbreaks from July 1994 to June 2002 [12], there is a similar geographic distribution of *N*. *meningitidis* disease activity in HealthMap. The distribution of cases captured for the school setting (57.8%) and the community setting (42.2%) by HealthMap alerts were similar to that reported previously in the setting of *N*. *meningitidis* outbreaks (44.9% and 36.2%, respectively) [12]. Fig 2 is a breakdown of state-specific HealthMap case alerts for *N*. *meningitidis* annually from beginning of 2010 to end of 2013 (2010 = blue, 2011 = orange, 2012 = light grey, and 2013 = dark grey). From this breakdown, it shows that 20 states represented the case alert activity captured by HealthMap during this four-year period. The top-two states, by year, comprising the majority of internet-based case alerts were Oklahoma/Colorado (12.0%/55.4%) in 2010, Colorado/Oregon (16.7%/26.2%) in 2011, Oregon/California (34.6%/32.1%) in 2012, and California/New Jersey (50.0%/32.6%) in 2013.

#### Accuracy of HealthMap capture of meningococcal disease

**Fig 3:**
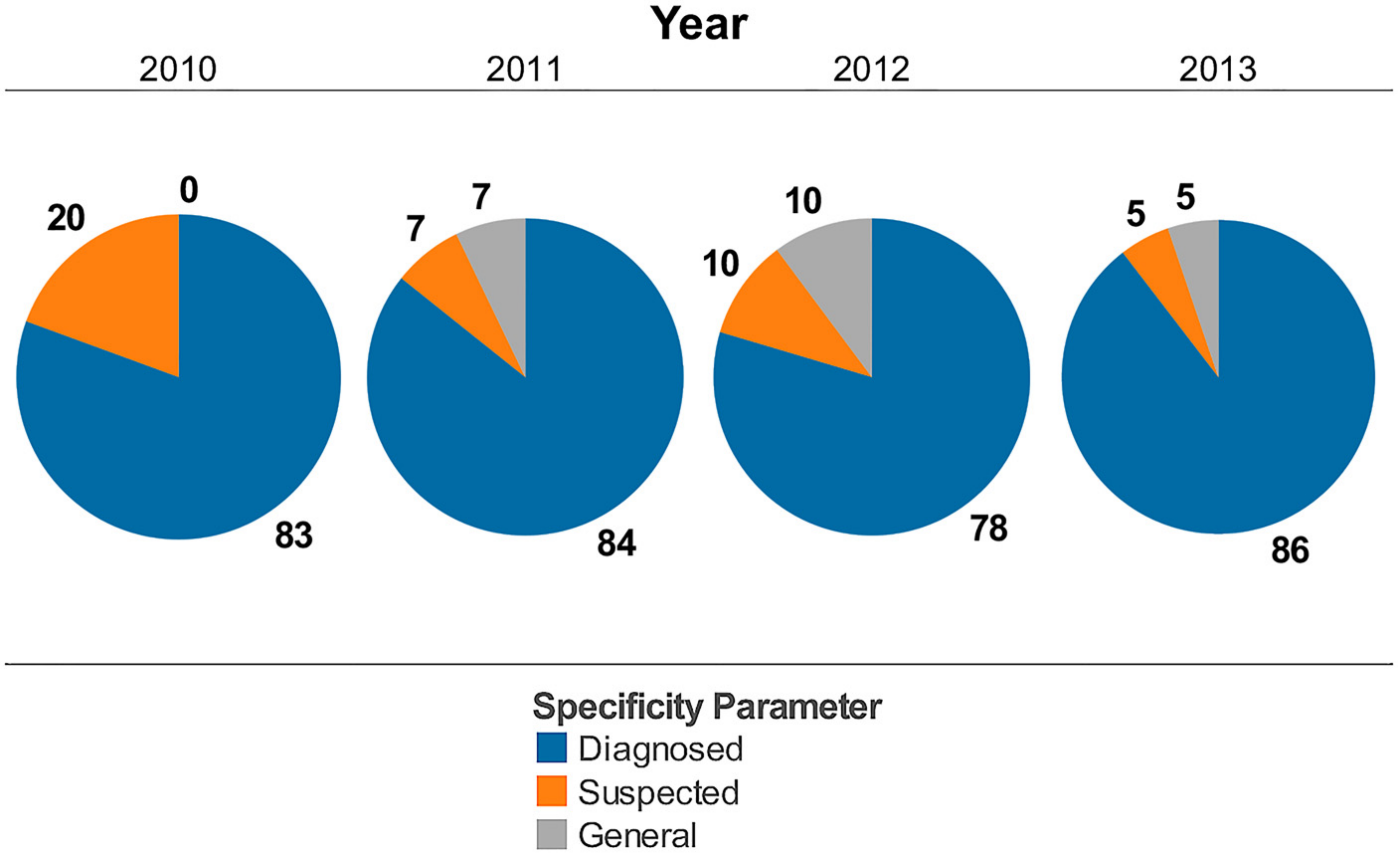
Specificity of HealthMap case alerts for actual *Neisseria meningitidis* disease activity from 2010–2013. The specificity, on average, for HealthMap alerts during 2010 to 2013 were as follows: 83.3% were diagnosed cases, 10.5% were suspected cases, and 5.6% were related news but not actual cases of *Neisseria meningitidis*. Pie graphs illustrate percentages for alerts per year with numerical values indicating actual number of alerts. Blue color indicates diagnosed *Neisseria meningitidis*, orange color indicates suspected *Neisseria meningitidis*, and light grey color indicates general information associated with meningococcal disease (related news articles but not actual cases).

Fig 3 includes information on the specificity of the disease cases captured by the HealthMap automated search algorithm. On average, 83.3% of reported cases identified by HealthMap represented diagnosed cases (blue color), 10.5% represented suspected cases (orange color), and 5.6% were related news but not actual cases of *N*. *meningitidis* (light grey color). Table 1 indicates the total cases captured through internet data sources utilized by HealthMap for 2010–2013 (30, 28, 22, and 40 cases, respectively) and illustrates that serogroup specific information for these cases was not available in 53.3%, 82.1%, 68.1%, and 52.5% of alerts.

**Table 1:** Meningococcal disease cases identified by HealthMap alerts.

Year	School (# of cases)	Community (# of cases)	Alive / Dead	Distribution
2010	Red Bluff, CALIFORNIA (1)	Red Bluff/Tehama County,	16/14	Total = 30 cases
2010	Denver/Fort Collins, COLORADO	CALIFORNIA (1B+1)		B = 23%
	(3C+1)	Larimer County, COLORADO		C = 23%
2010	Clare County, MICHIGAN (1)	(4C+1B)		Y = 0%
2010	Long Island, NEW YORK (1)	Bullitt County, KENTUCKY (1)		? = 54%
2010	Athens/Franklin County, OHIO (4B)	Franklin County, OHIO (1B)		
2010	Oologah, OKLAHOMA (7)			
2010	Roanoke College, VIRGINIA (1)			
2010	Madison, WISCONSIN (2)			
2011	Shasta County, CALIFORNIA (1)	Craig, ALASKA (1)	19/9	Total = 28 cases
2011	Boulder, COLORADO (1)	Fort Smith, ARKANSAS (1)		B = 4%
2011	Washington, DISTRICT OF	Los Angeles County,		C = 7%
	COLUMBIA (1)	CALIFORNIA (7)		Y = 7%
2011	Canton, NEW YORK (1)	Larimer County, COLORADO		? = 82%
2011	Columbus, OHIO (2)	(1Y)		
2011	Norman, OKLAHOMA (1)	Hillsborough/Broward		
2011	Prinville, OREGON (1C)	County, FLORIDA (2)		
2011	Bethlehem, PENNSYLVANIA (1)	Morgan County, OHIO (1)		
2011	Caroline County, VIRGINIA (1)	Bend/Prinville/Deschutes		
2011	Greenbay, WISCONSIN (1)	County, OREGON		
		(1C+1B+1Y+1)		
2012	Sacramento/San Francisco/	Clearlake/Ukiah, CALIFORNIA	14/8	Total = 22 cases
	San Diego, CALIFORNIA (3+1B)	(2)		B = 5%
2012	Tallahassee/Miami, FLORIDA (2)	New York, NEW YORK (4C)		C = 27%
2012	East Lansing/Traverse City,	Pawnee County, OKLAHOMA		Y = 0%
	MICHIGAN (2)	(1)		? = 68%
2012	St. Louis, MISSOURI (1)	Prinville/Eugene, OREGON		
2012	Prinville/Eugene, OREGON	(1C+2)		
	(1C+2)			
2013	Maricopa County, ARIZONA (1B)	Navajo/Maricopa County	34/6	Total = 40 cases
2013	Petaluma/San Diego/Shasta	ARIZONA (2B)		B = 43%
	County/Santa Barbara,	Huntington Beach/San		C = 5%
	CALIFORNIA (3+5B)	Diego/West Hollywood/		Y = 0%
2013	Chicago, ILLINOIS (1C)	Willits, CALIFORNIA		? = 52%
2013	South Bend/Muncie, INDIANA (2)	(6+1C)		
2013	Princeton/West Long Branch,	New York, NEW YORK (4)		
	NEW JERSEY (8B+1)			
2013	Wester Chester/Reading/			
	University Park,			
	PENNSYLVANIA (4)			
2013	Charlottesville, VIRGINIA (1B)			
2013	Madison, WISCONSIN (1)			

Example of community-identified and reported meningitis outbreaks in the United States in a four-year period. Based on the source of information and the stage of the outbreak, HealthMap is able to provide geographical and numerical information on cases and also displays information related to disease-causing serotype (when available).

### Epidemiology of vaccine-preventable serogroup ACWY versus serogroup B

**Fig 4:**
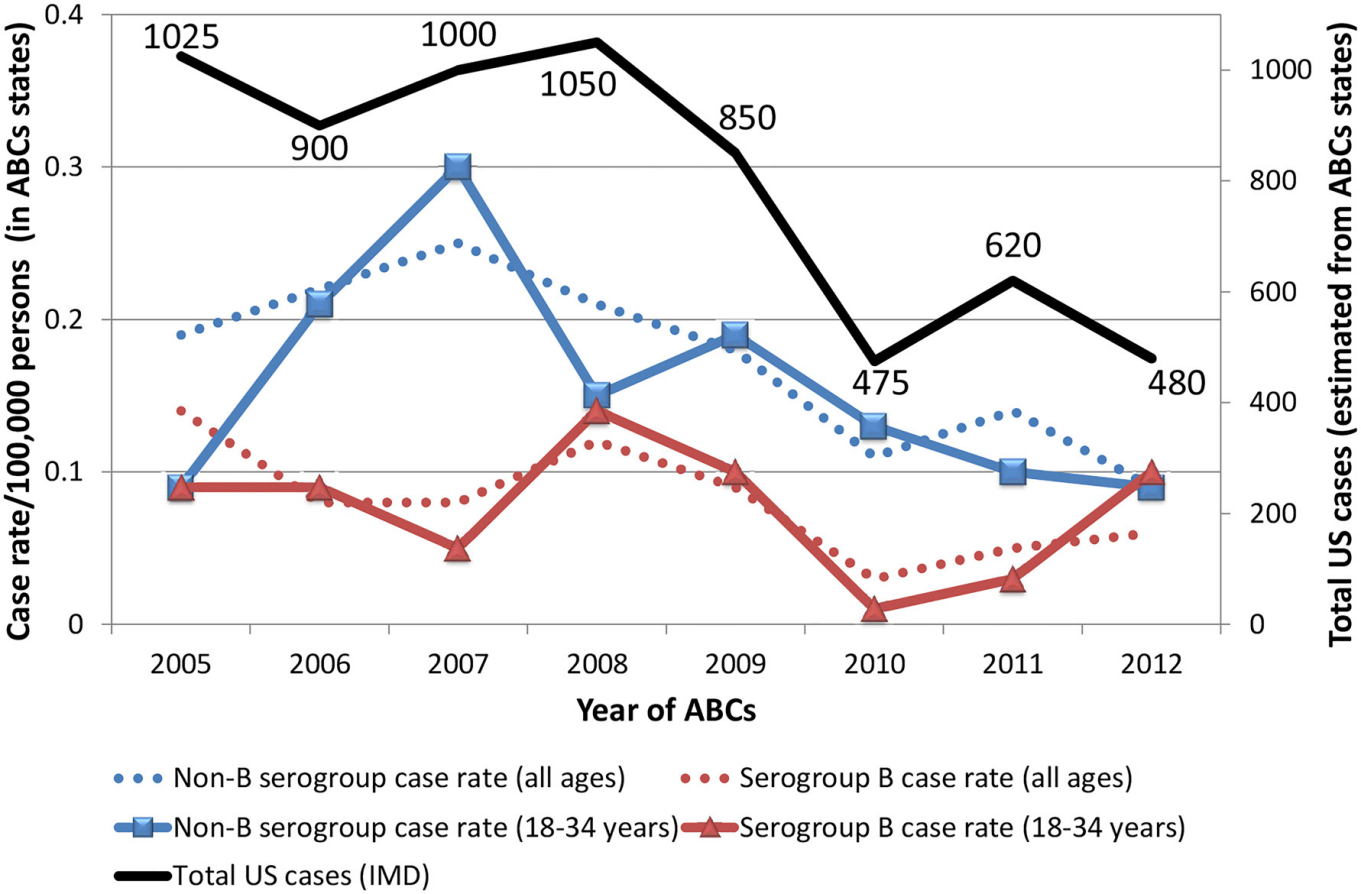
Invasive meningococcal disease (IMD) reported over the past eight years by Active Bacterial Core Surveillance. Data plotted from Active Bacterial Core Surveillance (ABCs) Emerging Infections Program Network (states highlighted in white). There is a decline of total cases (solid black line) in the ABCs states from 1,025 cases in 2005 to what appears to be a plateau ranging from 475 to 620 cases during 2010 to 2012. The dotted blue line indicates the decreasing trend in case rates per 100,000 persons (all age groups) for non-B serogroups from 2007 to 2012. The dotted red line indicates a more variable trend in case rates per 100,000 persons (all age groups) for the serogroup B that appears to show an increasing trend since 2010. This variability in case rate for serogroup B is expected since the MenACWY vaccine introduced in 2005 would not be protective against serogroup B. No deviation from these trends occur when analyzing specifically case rates in 18–34 year old individuals (university age group) as indicated for non-B serogroups (solid blue line) and serogroup B (solid red line). Case rates for serogroup B meningococcus in children < 1 year of age are not shown but are oscillating at a much higher case rate than all other age groups with values between 1.0 to 2.9 cases per 100,000 population compared to non-B serogroups that range from 0.6 to 1.2 cases per 100,000 population.

**Fig 5:**
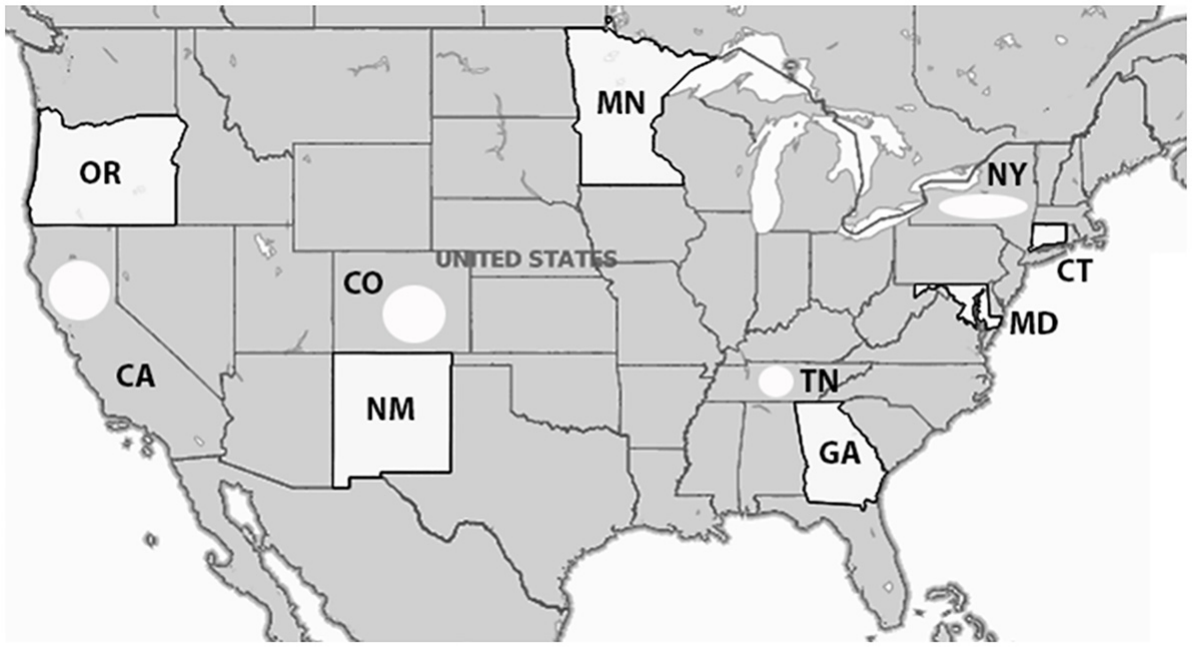
Population under suveillance by ABC for Neisseria meningitidis (adapted from CDC website). Non-B serogroup and serogroup B case rates reflects ABCs figures in ten US states (white shading of entire state indicates that total state population under surveillance while white shaded circle indicates that population of certain counties within the state are under surveillance). Numbers of inhabitants being followed per state are as follows: CT = 3,590,347; GA = 9,919,945; MD = 5,884,563; MN = 5,379,139; NM = 2,085,538; OR = 3,899,353; TN = 3,872,994 (20 urban counties); CA = 3,460,180 (3 county Bay area); CO = 2,532,982 (5 county Denver area); NY = 2,178,020 (7 county Rochester area and 8 county Albany area). Total population under surveillance by ABCs is 42,803,061.

Fig 4 illustrates the trend in meningococcal cases from 2005 to 2012 using ABCs surveillance data obtained from the CDC surveillance for *N*. *meningitidis* [11]. ABCs surveillance reflects ten US states representing 39 to 43 million inhabitants over eight years (Fig 5). There is a decline of total cases (Fig 4, solid black line) in the ABCs states from 1,025 cases in 2005 to what appears to be a plateau ranging from 475 to 620 cases during 2010 to 2012. The dotted blue line indicates the decreasing trend in case rates per 100,000 persons (all age groups) for non-B serogroups from 2007 to 2012. The dotted red line indicates a more variable trend in case rates per 100,000 persons (all age groups) for the serogroup B that appears to show an increasing trend since 2010. This variability in case rate for serogroup B is expected since the MenACWY vaccine introduced in 2005 would not be protective against serogroup B. No deviation from these trends occur when analyzing specifically case rates in 18–34 year old individuals (university age group) as indicated for non-B serogroups (solid blue line) and serogroup B (solid red line). Case rates from serogroup B meningococcus in children < 1 year of age were not included but are oscillating at a much higher case rate than all other age groups with values between 1.0 to 2.9 cases per 100,000 population compared to non-B serogroups that range from 0.6 to 1.2 cases per 100,000 population.

### Detecting HealthMap signals across national borders for emerging *N*. *meningitidis* threats

**Fig 6:**
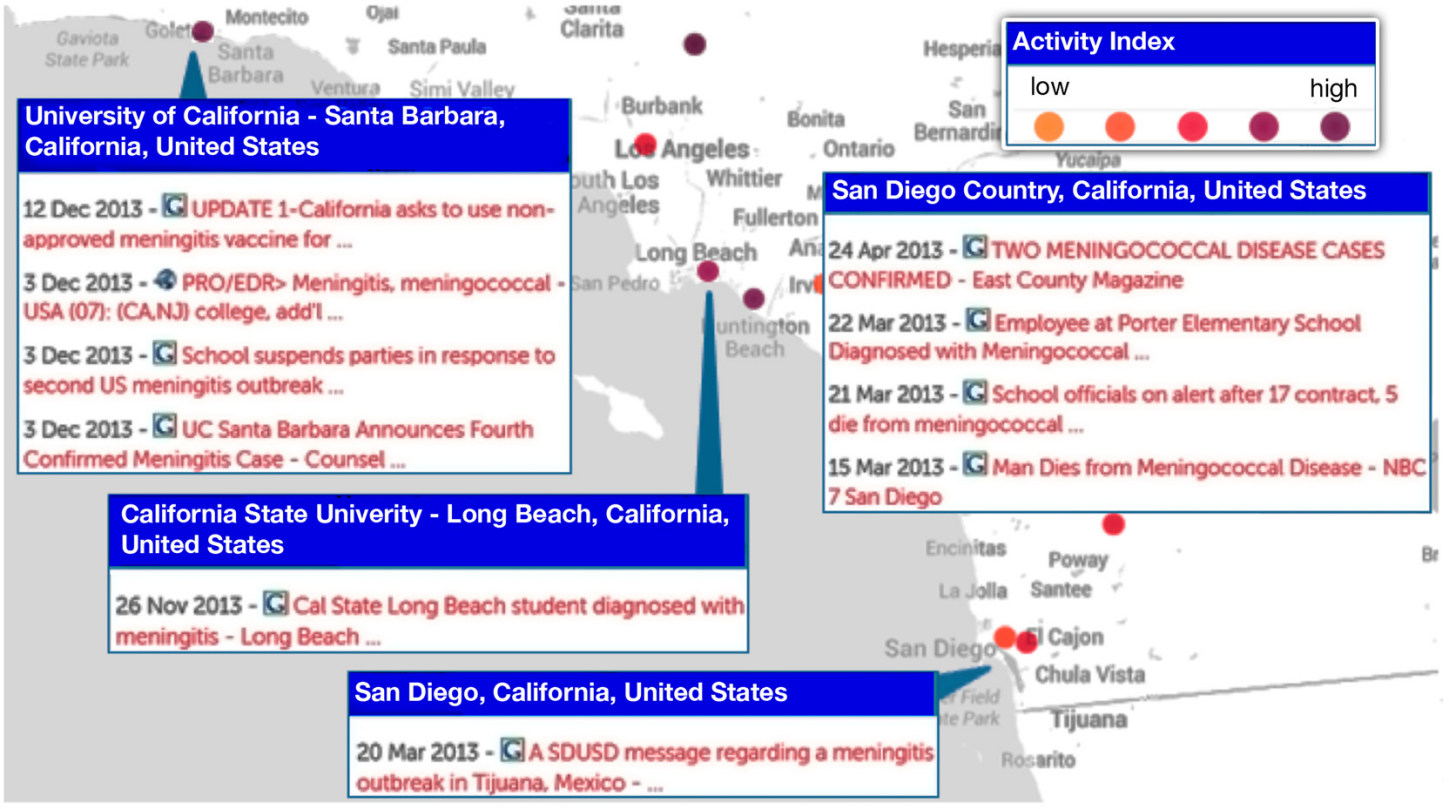
Local view of HealthMap data for Neisseria meningitidis alerts from 2010 to 2013. Multiple alerts in Southern California including one in San Diego refering to a meningitis outbreak across the border in Tijuana, Mexico. Marker color (light orange to darker purple) reflects increasing significance assigned by HealthMap users or news volume associated with the alert. Each marker may represent more than one *Neisseria meningitidis* alert (as seen in the break-out box). What becomes readily apparent from the image is the proximity of Tijuana to the southern border of the United States. In the first three months of 2013, there had been 17 cases (including six deaths) in Tijuana. Individuals today are more mobile than before which underscores the relevance of disease activity awareness in cities bordering countries.

**Fig 7:**
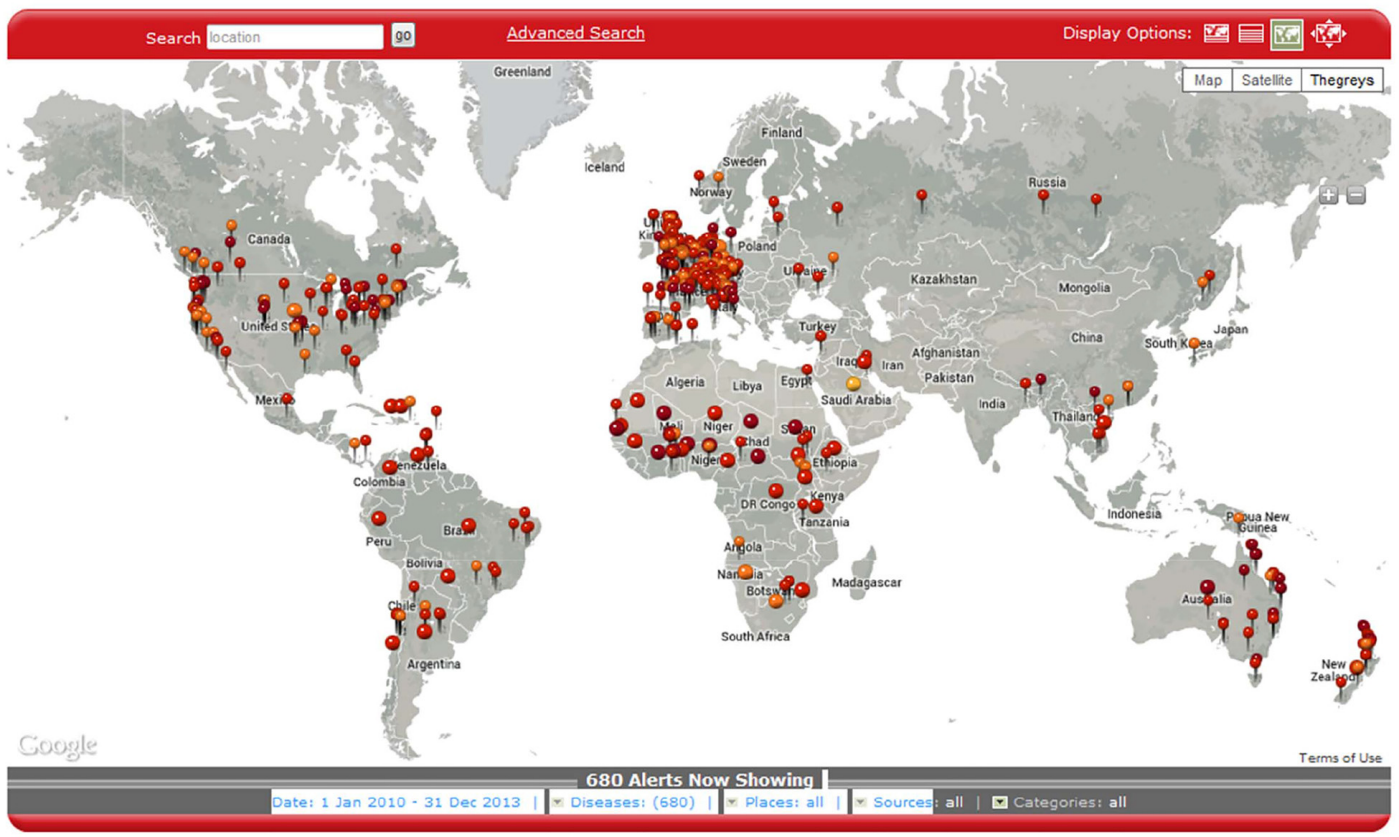
Global view of HealthMap data for Neisseria meningitidis alerts from 2010 to 2013. Multiple alerts globally including Neisseria meningococcus activity near the northern border of the US with Canada (cities in British Columbia and Ontario). Marker color (light orange to darker red) indicates increasing newsworthiness. Smaller marker size reflects local alert for city, state, or province while larger marker size reflects a country-level alert. Of the eight cases captured by HealthMap in Canada between 2010 to 2013, the bordering cities reported one case in a student in Western Ontario (2010), one death in a student in Hamilton (2010), and one death in a student in Vancouver Island (2012). HealthMap disease activity across borders could contribute to understanding disease transmission and subsequent outbreaks.

Fig 6 is an illustration of a close-up view from HealthMap illustrating the multiple *N*. *meningitidis* alerts for Southern California for 2013 including one in San Diego referring to a meningitis outbreak in Tijuana, Mexico [13]. What becomes readily apparent from the image is the proximity of Tijuana to the southern border of the United States. In the first three months of 2013, there had been 17 cases (including six deaths) in Tijuana [13]. If the Tijuana outbreak cases were counted as US cases, this would increase the total number of cases for 2013 from 40 to 57 patients (Table 1). In Fig 7, HealthMap is illustrating *N*. *meningitidis* disease alerts globally and illustrates *N*. *meningitidis* disease activity near the northern border of the United States with Canada (involving cities in British Columbia and Ontario). Of the eight cases captured by HealthMap in Canada between 2010 to 2013, the bordering cities reported one case in a student in Western Ontario (2010), one death in a student in Hamilton (2010), and one death in a student in Vancouver Island (2012).

### “Connecting the dots” to disease transmission using HealthMap

**Fig 8:**
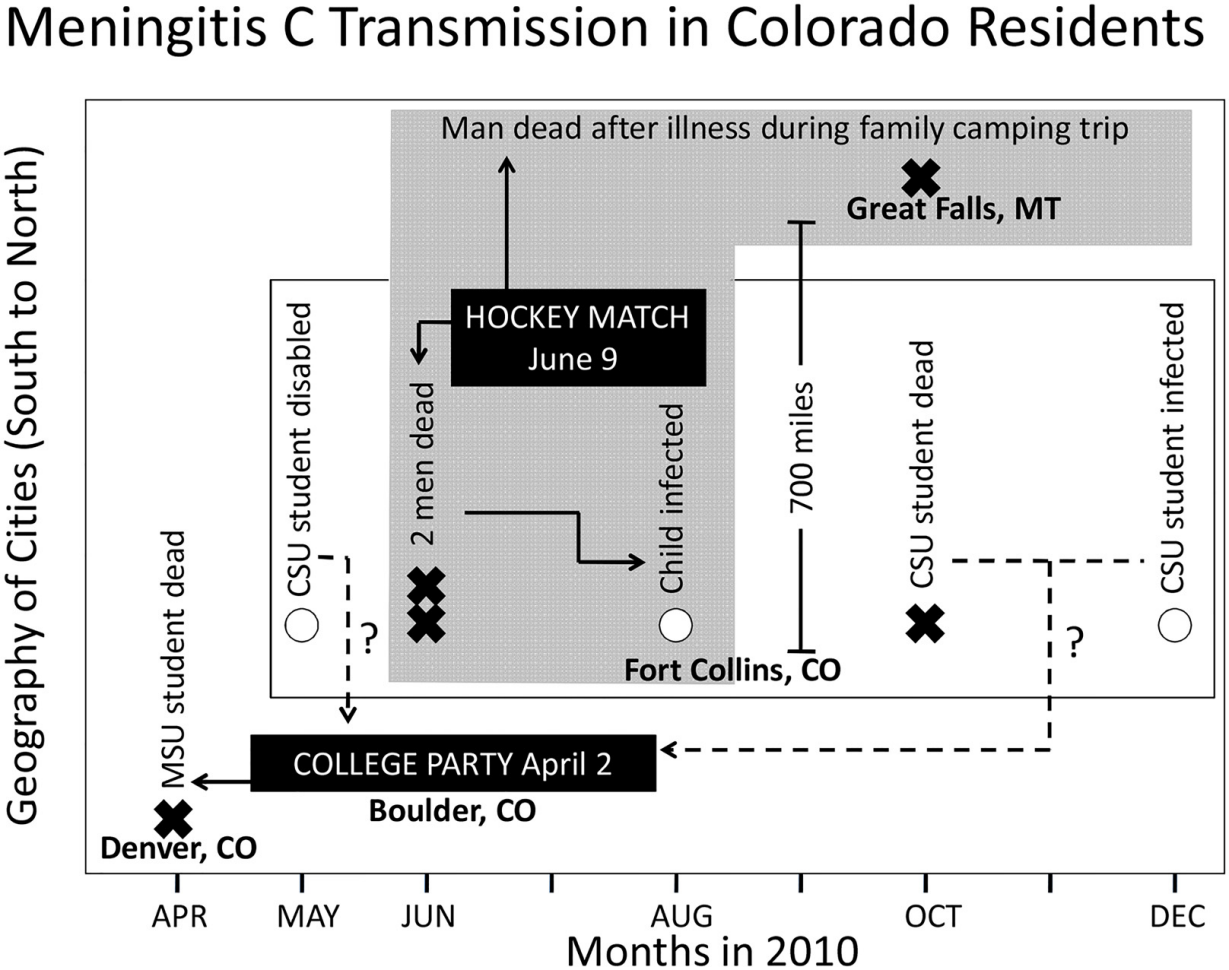
Understanding disease transmission from disease alerts in HealthMap. The figure illustrates on the y-axis the geographic location from south to north and on the x-axis, monthly intervals between April and December of 2010. The circles indicate survivors of meningococcal infection while the cross-marks indicate deaths. Data captured from real-time informatics trace eight subjects infected with Neisseria meningococcus back to possible disease initiation at a college party in Boulder, Colorado, with subsequent amplification at a hockey match in Fort Collins, Colorado, two months later.

Fig 8 is an example of how real-time informatics can be used to connect what appear to be unrelated meningococcal disease cases (due to serogroup C) that differ by state, city, site, and time. The data indicate one death in April (Metropolitan State University student in Denver, Colorado), two survivors and one death between May and December (Colorado State University students at Fort Collins, Colorado), two deaths and one survivor between June and August (two men and one child in Fort Collins, Colorado), and one death in October (a man in Great Falls, Montana). Using the news data captured with HealthMap, what becomes apparent is that the disease transmission may have started at a college party in Boulder, Colorado attended by the student from Denver who died. This party was also attended by Colorado State University (CSU) students from Fort Collins. While it is not clear whether the three CSU students infected with meningococcus serogroup C attended the party in Boulder, the infection in one of the three students predated the infection resulting in the death of two men and the hospitalization of one child in Fort Collins. The infected child was related to one of the two men who died. These two men were players in a hockey match in June (which CSU students attended), and the man dying four months later in Great Falls, Montana was also a player in this hockey match.

## Discussion

### Real-Time “snapshot” of meningococcal disease activity by HealthMap

Surveillance monitoring systems based on Internet news can provide new dynamics to public health data analysis and, as illustrated in the 2009 A/H1N1 influenza pandemic, help to overcome certain limitations of existing public health surveillance systems including reporting delays, inconsistent population coverage, and poor accuracy in detecting emerging diseases. The current university-linked serogroup B outbreaks in 2013–2014 and the serogroup B outbreaks described previously in Oregon from 1994–1996 and in Ohio in 2010 [14] highlight the unpredictable nature of *N*. *meningitidis* outbreaks and the challenges facing public officials who need to rapidly identify whether an outbreak is occurring and trigger the necessary intervention to treat and prevent disease transmission. Given the historical data available for comparison and the recent advancement in the capabilities of informatics, we used HealthMap, an automated text processing system that collects data from diverse online sources including media reports and health department press releases, to detect cases of meningococcal disease and potential outbreaks in the US from 2010 to 2013. HealthMap application to emerging diseases was previously proven during the global surveillance monitoring of H1N1 influenza pandemic in 2009 [5]. This real-time informatics system captured a national distribution of meningitis cases (Fig 1) and increased prevalence for school-based settings (Table 1) similar to what has been published in an older US study using traditional and more comprehensive surveillance [12]. While the Internet-based data streaming approach used by HealthMap underestimates US disease burden (Table 1) when compared to estimates from ABCs (Fig 4), it is a very rapid method for acquiring a snapshot of meningococcal disease visibility as a function of time (Figs 2 and 3). Predicting when a sporadic case will evolve into an outbreak of the proportions seen at Princeton, New Jersey, and Santa Barbara, California, remains problematic with traditional surveillance and could be bridged by the application of real-time informatics such as HealthMap.

### Detailed epidemiology captured by traditional surveillance systems

The real-time informatics approach used by HealthMap has been active since 2006 and its capacities have evolved significantly for disease monitoring [15–22] with the addition of data capture for *N*. *meningitidis* being fully operational as of 2010. However, internet-based data streaming cannot yet provide the level of epidemiological detail captured by traditional surveillance approaches which have also investigated patterns in meningococcal disease historically. Traditional surveillance has demonstrated that, since 1991, the frequency of local meningococcal outbreaks has increased with the majority of cases being due to serogroup C [23]. Since 1997, local outbreaks due to serogroups Y and B have also been reported [23]. A study published in 2006 characterized outbreak-associated and sporadic cases in the US during an eight-year period from 1994 through 2002 and identified 69 outbreaks (3–14 outbreaks per year) involving 229 patients from 30 states [12]. For all outbreaks, serogroup C was predominant (66.4%) followed by B (23.6%), Y (10.0%), and W (0%). During the same period, sporadic cases were predominantly caused by serogroup B (36.7%), followed by Y (32.5%), C (28.6%), and W (2.2%) [12]. This 2006 publication [12] concluded that 1) outbreak-associated cases were more likely to die compared to sporadic disease cases, 2) outbreak-associated cases most often involved serogroup C, and 3) institutional outbreaks generally last less than two weeks.

However, in the case of the 2013 outbreaks involving Princeton University and University of California Santa Barbara, the serogroup involved was meningococcus B and these institutional outbreaks persisted for months as captured by HealthMap data-streaming. These recent outbreaks suggests a change in meningococcal serogroup distribution which could be expected due to the introduction of vaccines protective against A, C, W, and Y (decreasing incidence of non-serogroup B meningococcus captured by ABCs in Fig 4). Since the peak of meningococcal disease in the 1990s in the US, disease incidence has decreased to 0.2 cases per 100,000 population in all age groups in 2011 [24]. The burden of disease is highest among infants younger than 1 year of age (2.6 cases per 100,000 persons), adolescents 16 to 21 years of age (0.4 cases per 100,000 persons), and adults older than 65 years of age (0.3 cases per 100,000 persons) [24]. While serogroups B, C, and Y are each responsible for approximately one third of meningococcal cases in the US, the proportion caused by each serogroup varies by age group. For example, 60% of disease in children younger than 60 months of age is caused by serogroup B. However, 73% of disease in persons aged ≥ 11 years is caused by serogroup C, Y, or W. While no serogroup B vaccine is licensed in the US for children younger than 60 months of age, quadrivalent meningococcal glycoconjugate vaccines protective against serogroups A, C, W, and Y (MenACWY) have been licensed in the US since 2005 [24]. MenACWY vaccine coverage has increased to over 70% by 2011 in adolescents 13 to 17 years of age [24] with the estimated number of annual cases from serogroups C and Y decreasing by 75% in individuals 11 to 14 years of age from 2000–2004 to 2005–2009 [23]. However, for individuals 15 to 18 years of age, serogroup C and Y cases have decreased only by 27% and a peak in meningococcal disease has persisted among persons aged 18 to 21 years [23].

### Using informatics to put into context emerging *N*. *meningitidis* disease threats and related public health interventions

The ability of informatics-based approaches to filter through local, national, and global disease activity in real-time provides another feature not readily apparent from traditional surveillance approaches—modes of disease transmission. Individuals today are more mobile than before which underscores the relevance of disease activity awareness in cities bordering countries. From Fig 7, it becomes apparent that HealthMap disease activity across borders could contribute to understanding disease transmission and subsequent outbreaks. As an example, the HealthMap alerts for Southern California in 2013 brought to attention a meningitis outbreak in Tijuana, Mexico (Fig 6). According to the San Diego Deputy Public Health director, the outbreak in Tijuana in 2013 was three times the incidence seen in Tijuana annually [13]. Tijuana is the largest city in the Mexican state of Baja California and is situated on the US-Mexico border adjacent to San Diego, California. This is the most traversed international border in the world [25] with the two border crossing stations in Tijuana accounting for an estimated 300,000 border crossings each day. Notably, a subsequent epidemiological study on meningococcal disease in Latin America from 1945 to 2010 demonstrated that serogroup A has virtually disappeared, and that serogroups B and C are responsible for most cases [26]. The disease activity captured by HealthMap for Canadian cities (Fig 7) near the US border reflected only two cases in 2010 and one case in 2012 that may be reflecting the timely incorporation of vaccines into the national schedules by Health Canada. The quadrivalent conjugated vaccine protective for A,C,W,Y was licensed in Canada in 2007 [27] and, more recently, a recombinant serogroup B vaccine was approved on January 2014 [28].

### *N*. *meningitidis* outbreaks: potential of internet-based data streaming to de-convolute disease transmission or capture intervention-related signals

The unpredictable nature of meningococcal outbreaks and the protracted timeline during which enough cases accumulate to trigger declaration of an outbreak were recently highlighted by the serogroup B meningococcus university-linked outbreaks that occurred on both the east and west coasts of the US in 2013. More recently, on March 10, 2014, a student at Drexel University died from serogroup B meningococcal disease. CDC laboratory analysis, based on genetic fingerprinting, demonstrated that the strain infecting this student matched that in the Princeton University outbreak. This student had been in close contact with students from Princeton University about a week before illness, and the laboratory analysis indicated that the outbreak strain may still be present in the Princeton community and require vigilance for additional cases [29]. These university outbreaks highlight the limitations of antibiotic chemoprophylaxis interventions that require the identification and treatment of all individuals at risk in order to avoid subsequent disease transmission by untreated carriers of subclinical disease. This was evidenced during a serogroup B outbreak in a hotel [30] and another in a middle school [31] in 1995. However, real-time capture of cases by internet-based informatics can complement traditional surveillance monitoring approaches leading to increasing confidence and resourcing in outbreak settings by decreasing time to outbreak declaration and medical handling. As illustrated in Fig 8, modes of disease transmission could be traced to ensure targeted administration of antibiotic therapy or prophylactic interventions to the relevant at-risk population. Finally, internet-based monitoring systems like HealthMap can provide a real-time channel for patients to report intervention-related failures or can be applied to safety data mining by scanning social media channels like Twitter and Facebook on a macro-level for patient-reported safety insights. This could include the direct reporting to regulatory authorities of medication or vaccine-associated adverse events identified through social media and mobile phone apps. These widely accessible and user-friendly computer-based options can support the tracking of subject safety and could facilitate decision-making related to the use of unlicensed therapies or vaccines in emergency situations. One such emergency situation was the Princeton University serogroup B meningococcal disease outbreak that began in the US in March 2013. An emergency vaccination campaign with an imported vaccine (Bexsero, approved in Australia, Europe, and Canada but not in the US) was able to be implemented in the US in December 2013 (approximately nine months later) only under special regulatory procedures after the CDC filed an Investigational New Drug application with the Food and Drug Administration requesting special permission for use [32].

With regards to invasive meningococcal disease (IMD), a potential limitation of using HealthMap may be related to the inability to identify and report the serotypes specifically linked to the disease outbreak. Traditionally, all US states have mandated reporting of IMD [33] and require health care providers to immediately report suspected cases to assure rapid identification of close contacts and administration of antibiotic prophylaxis [23]. However, despite the swift execution of the protocol established to confirm these reports, there is always a window of delay secondary to the specialized public health molecular and microbiological laboratory testing necessary to confirm cases in establish IMD clusters. Usually, during an outbreak, the high case rates increase awareness in health authorities, but options for implementing the best tailored, prophylactic measure requires understanding the extent and mode of transmission involved in that particular outbreak. A national network of state epidemiologists and CDC maintain regular communication on the outbreak status through electronic alerts over the secure CDC Epi-X system and direct communication between states when out-of-state exposures occur.

## Conclusions

International events with the potential to cross borders (e.g., 2009 H1N1 pandemic influenza) are probably the best example of the application of HealthMap and highlight its potential use for other emerging worldwide threats (e.g., SARS, avian influenza, or Ebola virus). The underreporting of disease cases by internet-based data streaming in specific *N*. *meningitidis* outbreak modes makes inadequate any comparison to epidemiological data captured by more comprehensive traditional *N*. *meningitidis* surveillance such as the ABCs network published by the CDC. However, the natural delays in compiling reports from traditional surveillance (at the time of writing, ABCs *N*. *meningitidis* data for 2013 is listed as being provisional) emphasize the utility of real-time internet-based data streaming for providing a real-time “snapshot” of meningococcal disease. In this investigation on surveillance of *N*. *meningitidis* disease activity and transmission using the HealthMap information tool, what becomes apparent is that this information technology can complement epidemiological data captured by traditional surveillance monitoring systems by quickly filling gaps (including the visualization of modes of meningococcal disease transmission in outbreaks) allowing for better resource and action planning. For the general public and the public health community, HealthMap surveillance of meningococcal disease activity can increase disease awareness by collecting an immense amount of disease/outbreak reports and putting them in a widely accessible and user-friendly platform. This user-friendly platform of HealthMap has the potential to additionally complement traditional surveillance monitoring by serving as an internet-based real-time channel for patients infected with *N*. *meningitidis* to report intervention-related failures.
